# Supplementary material for: ProteinShader: illustrative rendering of macromolecules
Source: BMC Struct Biol. 2009 Mar 30;9:19. doi: 10.1186/1472-6807-9-19 (PMC2672931; doi:10.1186/1472-6807-9-19)
Supplement: Additional file 1 — ProteinShader program without source code. This compressed file contains the complete ProteinShader program including associated libraries, but no source code. A README.txt file gives an overview of the ProteinShader distribution, and the index.html file in the help subdirectory has directions on getting started with the program as well as a set of tutorials. [file 1472-6807-9-19-S1.zip › ProteinShader-beta-0_9_4-binary/help/api/org/proteinshader/structure/enums/CPKColorEnum.html]

CPKColorEnum (ProteinShader API)


|  |  |  |
| --- | --- | --- |
| **Overview** \| **Package** \| **Class** \| **Use** \| **Tree** \| **Deprecated** \| **Index** \| **Help** | |  |
| **PREV CLASS**   **NEXT CLASS** | **FRAMES**    **NO FRAMES**     **All Classes** |  |
| SUMMARY: NESTED \| ENUM CONSTANTS \| FIELD \| METHOD | DETAIL: ENUM CONSTANTS \| FIELD \| METHOD |  |


---


## org.proteinshader.structure.enums Enum CPKColorEnum

```
java.lang.Object
  java.lang.Enum<CPKColorEnum>
      org.proteinshader.structure.enums.CPKColorEnum
```

**All Implemented Interfaces:**: Serializable, Comparable<CPKColorEnum>

---

``` public enum CPKColorEnum extends Enum<CPKColorEnum> ```

Provides an enumeration of the CPK color scheme that is needed by
AtomEnum. The CPK color scheme was developed by Corey, Pauling, and
Koltun for use by chemists and molecular biologists for coloring atoms
in molecular models.
